# Moderating effect of the environment in the relationship between mobility and school participation in children and adolescents with cerebral palsy

**DOI:** 10.1590/bjpt-rbf.2014.0127

**Published:** 2015-09-01

**Authors:** Sheyla R. C. Furtado, Rosana F. Sampaio, Renata N. Kirkwood, Daniela V. Vaz, Marisa C. Mancini

**Affiliations:** 1 Programa de Pós-graduação em Ciências da Reabilitação, Escola de Educação Física, Fisioterapia e Terapia Ocupacional, Universidade Federal de Minas Gerais (UFMG), Belo Horizonte, MG, Brazil

**Keywords:** functioning, participation, school, rehabilitation

## Abstract

**BACKGROUND:**

The literature demonstrates that the social participation of children with disabilities is influenced by both their functional skills repertoire and environmental factors. However, it is not yet known whether the effect of functional limitations on social participation is minimized or enhanced by the environmental facilitators and barriers. This study aimed to test this hypothesis.

**OBJECTIVE:**

To investigate the moderating effect of environmental factors in the relationship between mobility and school participation of children and adolescents with cerebral palsy (CP).

**METHOD:**

Participants were 102 elementary school children and adolescents with CP, aged 6 to 17 years, classified as levels I, II, and III according to the Gross Motor Classification System, along with their parents or caregivers and teachers. School participation and parents' perceptions of barriers were evaluated using the School Function Assessment and the Craig Hospital Inventory of Environmental Factors (CHIEF), respectively.

**RESULTS:**

The regression model failed to reveal a moderating effect of environmental factors in the relationship between mobility and school participation. While mobility was a strong predictor of participation, environmental factors demonstrated a weak predictive effect on the latter. The CHIEF subscale school/work showed the factors which were greatest barrier to children's participation, while the subscale attitude/support had the least impact.

**CONCLUSION:**

The absence of moderation on the tested relationship suggests that, when investigated under the negative perspective of environmental barriers, the contextual factors do not modify the relationship between mobility and school participation. Factors specific to the school environment might add to the present study's results regarding the effect of school participation in this population.

## Introduction

In recent years, efforts have been made to understand the environmental impact on the lives of people with disabilities^1^^-^ 7. Understanding the relationships established between individuals and their context is consistent with the International Classification of Functioning, Disability and Health (ICF) model^8^. According to this model, the interaction between individuals with health conditions and their context, represented by personal and environmental factors, may affect functioning and disability components, including participation^8^.

Social participation refers to involvement in daily life situations^8^, enabling individuals to build their relationships and to develop skills and competencies for meeting the social demands, thus allowing them to find purpose and meaning in life^9^. The literature on the participation of children with disabilities provides evidence of the impact of environmental factors on functioning and disability processes^1^^-^ 7. Children with similar types of cerebral palsy (CP) living in places with facilitating disability services and structures have been found to have higher participation scores^7^. Furthermore, children with CP with the same motor function classification showed different patterns of mobility in different contexts^6^. Parents perceived factors such as lack of social support, negative attitudes, and inadequate physical environment as barriers to the participation of their children in school^10^. This evidence identifies the elements that restrict the social participation of children with disabilities, unraveling the relationship between environment and function.

The mobility repertoire of children is also a relevant variable for the social participation of children with physical disabilities^11^^-^ 14. Children with CP who walk without the use of orthoses showed better performance in daily life activities and social participation than children who use wheelchairs^13^. Similarly, Kerr et al.^12^ observed a significant association between motor function and social participation, demonstrating that physical independence is associated with a lower restriction on the participation of children with CP.

The relationship between contextual factors, mobility and social participation in children with CP has been examined. After using electric wheelchairs, children with spastic quadriplegia have become more independent in terms of mobility and have expanded their participation^15^. That result indicates that although the variables related to the repertoire of functional abilities and environmental factors individually affect the participation of children with disabilities, together, they may modify and enhance their effects.

Although the relevance of contextual factors was established in the ICF model, the description of those factors in the scientific literature has focused on identifying the contextual factors that act as barriers or facilitators^16^. However, given that the unit of analysis of the conceptual framework of the ICF is characterized by the individual-environment interaction, contextual factors play a central role in the disability and functioning processes. Thus, the effect of context should not be understood as a variable that individually affects the functioning components, but rather as an integral part of the interactive structure that characterizes the multidimensionality and complexity of disability and functioning processes. Contextual factors may play a moderating role in the dynamic structure of the different ICF domains, which is more in tune with the interactive nature of the model. Based on the conceptual ICF framework, the environmental factor may be considered a moderating factor when the relationship between the components activity and participation is significantly modified by their presence. This study aimed to examine the moderating effect of environmental factors in the relationship between mobility and school participation of children and youths with CP.

## Method

### Participants

The study sample included children and youths with CP, their parents or guardians, and their teachers. The sample size calculation (estimated n=99) was based on a study examining the school participation of children with CP with different levels of mobility^17^. The inclusion criteria were as follows: having been diagnosed with CP, aged from 6 to 18 years, walking with or without lower limb orthoses and possibly using a wheelchair for long distances, and being enrolled in elementary school. No sample loss occurred in this study. The sample was recruited from a children's rehabilitation center, the *Associação Mineira de Reabilitação* (AMR). The guardians of the children and youths signed a consent form approved by the Research Ethics Committee of the *Universidade Federal de Minas Gerais* (UFMG), Belo Horizonte, state of Minas Gerais (MG), Brazil (Opinion ETIC 028/09), allowing their participation in the study. They also signed a form consenting to the researcher contacting their child's school to administer the School Function Assessment questionnaire*.*

### Instrumentation

#### Gross Motor Function Classification System (GMFCS)

The GMFCS aims to assess the level of mobility based on the gross motor function limitations of children with CP^18^^,^ 19. Level I of the GMFCS represents children with no limitations, and level V represents children with the greatest mobility impairment. The difference between intermediate levels reflects functional constraints and the need for help, support and/or assistive technology.

#### School Function Assessment (SFA)

The SFA quantifies the functional performance of children with disabilities in a school environment and contextual factors into three domains: participation, task support, and activity performance. Domain I was used in this study, which evaluated the participation of students in six school contexts: classroom, playground, transportation to/from school, bathroom use, transitions in the classroom and between school environments, and school meals. The scores of each context ranged from one (extremely limited participation) to six (full participation). The raw score of each child was transformed into a criterion score, which ranged from 0 to 100. The SFA has demonstrated adequate psychometric qualities^20^.

#### Craig Hospital Inventory of Environmental Factors (CHIEF)

The Portuguese version of the CHIEF was used to evaluate the perception of parents of the impact of environmental barriers on the social participation of their children with CP^21^.

The CHIEF evaluates five domains/barriers: attitude and support, services and assistance, physical structure, policy and work and school. Each item is scored according to the frequency of perceived barriers and their magnitude. The CHIEF has three scoring methods for each item: the frequency score, which ranges from 0 to 4; the magnitude score, which ranges from 0 to 2; and the frequency-magnitude score, which is the product of the frequency and magnitude and ranges from 0 to 8. The total score is calculated by averaging the frequency, magnitude and frequency-magnitude scores of all items answered. In this questionnaire, higher scores indicate greater perception of environmental barriers. The instrument showed good reliability and good content, construct and discriminant validities^22^ and has been used in different populations^10^^,^ 22 ^-^ 24.

Furthermore, the demographic data of children/youths with CP and respondents, and the socioeconomic characteristics of families were collected using the Brazilian Economic Classification Criterion - Brazilian Association of Research Companies (Critério Classificação Econômica Brasil - Associação Brasileira de Empresa de Pesquisa, ABEP)^25^. That criterion consisted of a structured questionnaire with items on the presence and number of household items the family owns, and the education level of the household head. The sum of the item scores resulted in a total score, which could be converted into different economic class strata for families (A1, A2, B1, B2, C1, C2, D, E)^25^.

### Procedure

Initially, the guardians of the children and youths with CP answered the ABEP and CHIEF questionnaires through interviews, and the participants with CP were then classified based on their level of mobility as measured by the GMFCS. Three raters performed these evaluations in a rehabilitation center. An appointment was scheduled at the school to evaluate school participation so that the teacher could provide information on student performance. This questionnaire was administered by a single researcher.

The interrater correlation coefficient of the total frequency-magnitude score of the CHIEF ranged from 0.73 to 0.97, indicating good reliability indices. The interrater reliability of the GMFCS, evaluated using the quadratic kappa, was ≥0.98.

### Statistical analysis

Normality tests confirmed the normal distribution of the data. Descriptive analyses included Student's *t*-test to compare the difference between genders in the participation scores, and a one-way analysis of variance (ANOVA) was used to compare the differences between levels of the GMFCS and economic classes in the same outcome. The *post-hoc* (Tukey) test identified bivariate differences. Pearson's correlation analysis evaluated the relationships between the total frequency-magnitude score of the CHIEF and the participation score, and between the CHIEF subscales and the participation score.

Regression analysis tested the moderating effect of the CHIEF on the relationship between mobility as measured by the GMFCS and school participation as measured by the SFA of children and adolescents with CP through the interaction effect. The bivariate association between the independent (GMFCS) and moderating (CHIEF) variables and the dependent variable (participation) was initially tested. Based on the identification of significant associations, the regression model with stepwise entry of independent variables identified the group of variables that best explained the outcome of participation and the order of entry. The moderation test required multiplying the independent variable (GMFCS) by the moderating variable (CHIEF). The interaction product should have a significant effect on the regression model for moderation to occur. The significance level was set at 0.05 for all analyses. The data were analyzed using the Statistical Package for Social Science (SPSS Inc, Chicago, IL, USA) software, version 15.0.

## Results

Descriptive data of children and adolescents with CP, stratified according to the GMFCS level, and of their parents are shown in Table 1.

**Table 1.** Descriptive characteristics of children and adolescents with cerebral palsy and parents or guardians according to the motor function levels of the Gross Motor Function Classification System (GMFCS) (N=102).

Descriptive Variables		GMFCS		
		Level I	Level II	Level III
Children and adolescents with CP				
Number of participants		33	36	33
Age^*^	years	9.88 (2.82)	10.11 (2.65)	9.97 (2.92)
Sex^**^	F	19	16	13
	M	14	20	20
Education^*^	years	3.48 (2.12)	3.78 (2.09)	3.42 (2.41)
Parents or caregivers (respondents of CHIEF)				
Age^*^	years	41.45 (10.29)	38.56 (8.08)	38.83 (8.10)
Sex^**^	F	30	33	28
	M	3	3	5
Education^*^	years	8.70 (4.47)	8.33 (4.01)	9.91 (4.16)
CECB^**^	A1 and A2	4	2	5
	B1 and B2	3	5	6
	C1 and C2	20	24	19
	D	6	5	3

Chief = Craig Hospital Inventory of Environmental Factors. CECB = Criterion for Economics Classification Brazil (average family income in R$): A1 and A2 = from 9,733.00 to 6,564.00, B1 and B2 = from 3,479.00 to 2,013.00, C1 and C2 = from 1,195.00 to 726.00 and D = 485.00); sex (F=female, M=male). *Numbers indicate means and standard deviations in parentheses. **numbers indicate frequency of children/adolescents and respondents to the CHIEF at each level of the GMFCS.

**Figure 1.**
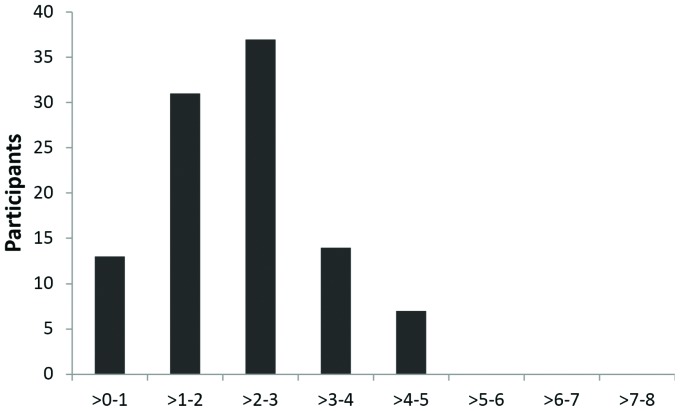
Frequency distribution of participants according to the Craig Hospital Inventory of Environmental Factors' (CHIEF's) frequency-magnitude score (N=102).

**Figure 2.**
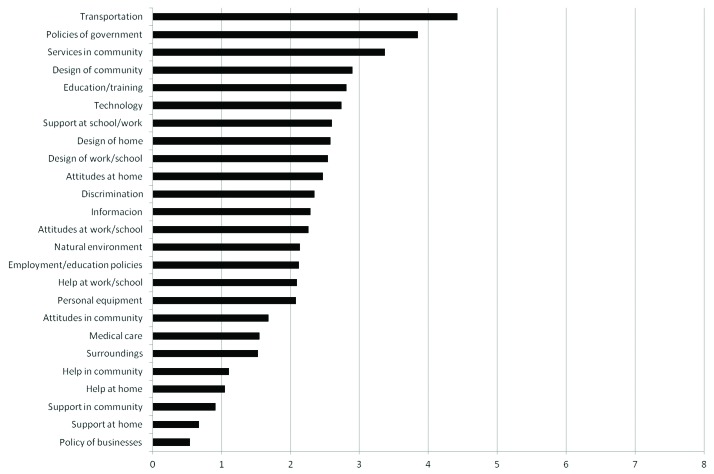
Means of the frequency-magnitude scores of each Craig Hospital Inventory of Environmental Factors (CHIEF) item in ascending order (scale 0-8).

The frequency distribution of the CHIEF frequency-magnitude scores (Figure 1) shows that 67% of respondents scored higher than one and lower than three on a scale from zero to eight. Transportation, government policy, and services in the community were identified by parents as the main barriers, while business policies and support at home and in the community were the items identified as the smallest barriers to the participation of their children (Figure 2).

**Figure 3.**
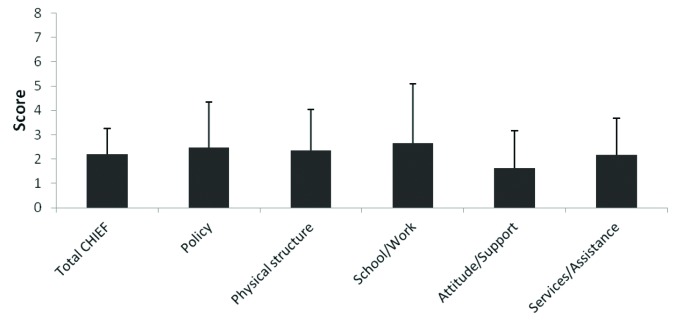
Means and standard deviations of frequency-magnitude scores from the subscales and total Craig Hospital Inventory of Environmental Factors (CHIEF) scores.

The school/work subscale of the CHIEF test, which refers to items related to assistance, attitude and support from people in that context, imposed the greatest barrier to the participation of their children with CP. The attitude and support subscale, which encompasses items related to attitude and support at home and in the community and to discrimination, was identified as the smallest barrier (Figure 3).

One hundred and two schools, representing all administrative districts of Belo Horizonte and eight municipalities, were visited to administer the SFA. The means and standard deviations of the CHIEF subscales and the six SFA contexts according to the motor function levels of the GMFCS are outlined in Table 2. The interviewed teachers have taught, on average, 15.27±8.7 years in regular classes and 5.10±5.20 in inclusive classes.

**Table 2.** Means and standard deviations of the subscales of the Craig Hospital Inventory of Environmental Factors (CHIEF) and the School Function Assessment (SFA) according to motor function levels of the (GMFCS) (N=102).

Variables	GMFCS		
	Level I (N=33)	Level II (N=36)	Level III (N=33)
Subscales of the CHIEF			
Policies	2.05±1.69	2.58±2.01	2.73±1.91
Physical structural	1.64±1.23	2.31±1.70	3.09±1.79
School/work	2.20±2.17	3.63±2.80	2.00±2.01
Attitude and support	1.79±1.56	1.66±1.34	1.47±1.74
Service and assistance	1.89±1.81	2.12±1.25	2.49±1.45
Total score (FxM)^*^	1.88±1.17	2.35±0.97	2.39±1.01
SFA			
Regular classroom	4.48±1.25	4.11±1.30	2.94±1.22
Playground	4.94±1.37	3.83±1.68	2.45±1.25
Transportation	5.06±1.32	4.33±1.24	1.64±1.11
Bathroom	5.58±0.83	4.94±1.22	2.58±1.32
Transition	5.33±0.92	4.64±1.22	2.82±1.26
Mealtime	5.52±0.76	4.83±1.08	3.55±1.37
SFA total	30.88±4.18	26.69±5.78	15.97±5.62
Criterion score	75.48±11.64	65.00±11.89	42.18±14.18

*Mean of frequency-magnitude score.

The Pearson's correlation test revealed a weak and negative association of the participation score and the total frequency-magnitude score of the CHIEF (*r*=-0.224; *p*=0.024), particularly with the subscales physical and structural (*r*=-0.326; *p*=0.001) and services and assistance (*r*=-0.281; *p*=0.004)^26^. ANOVA revealed differences in the participation score for the different GMFCS levels (*F*=60.43; *p*=0.0001). Specifically, differences were identified between levels I and II (*p<*0.0001), levels I and III (*p*<0.0001) and levels II and III (*p*<0.002). Children/adolescents with lower mobility impairment had higher participation. No significant differences were detected in the participation score regarding gender (*t*=-0.452; *p*=0.652) and social class (*F*=0.278; *p*=0.841).

Regression analysis indicated that the GMFCS variable explained 55% (F=60.43; p<0.0001) of the variability in the school participation score. The participation score decreased by 10.48 points in that model when changing from level I to level II of the GMFCS, and an even greater reduction, of approximately 30 points, occurred when comparing children/adolescents from level I with those from level III of the GMFCS. Such results show that mobility severity is a determining factor for the participation of children in school.

The CHIEF variables (frequency-magnitude score) and their subscales physical and structural barriers, service and assistance, which showed slight increases in the coefficients of determination (*R* 2=0.562, F=41.89, p<0.0001; *R* *2*=0.556, F=40.83, p<0.0001 and *R* *2*=0.576, F=44.45, p<0.0001, respectively), were individually added following the entry of the GMFCS in the model. However, in the presence of GMFCS, only the subscale service and assistance increased the prediction of school participation (b=-2.02; *p*=0.015). However, statistical significance was no longer observed when the interactions of the subscale service and assistance with the GMFCS levels were examined, which indicated that the effect of that subscale on participation was independent of the severity of the GMFCS (i.e., there was no moderating effect).

## Discussion

The results of this study showed that the perception of barriers and mobility variables individually affected the participation of children and adolescents with CP. Although the ICF conceptual framework proposed complex interactions between the functioning components and contextual factors, the purpose of this study of assessing the complexity of those relationships through moderation analysis showed no significant effect.

The variable mobility showed a strong association with the school participation of children and youths with CP, explaining more than 50% of its variability. The results indicated that children and adolescents with GMFCS level I CP had higher school participation scores than those with levels II and III CP. Similarly, differences were revealed between levels II and III, and those classified in level III had significantly lower participation scores. Corroborating those results, Schenker et al.^17^ showed that children with GMFCS level II CP who were included in either regular school classes or special classes had higher school participation scores than level III children. In another study, the Schenker et al.^9^ observed that the variable performance of children with CP during primarily physical school tasks had higher predictive power of school participation than performance on cognitive-behavioral tasks^9^. The scientific literature also showed the positive relationship of motor skills with the greater participation of children and adolescents with disabilities in leisure^14^^,^ 27, community^28^ and physical^27^^,^ 29 activities.

Unlike mobility, in this study, the perception of barriers had a small impact on the school participation of children and youths with CP. Similarities regarding the modest contribution of environmental factors to the participation of people with disabilities have also been reported by other authors^24^^,^ 30 ^,^ 31. Whiteneck et al.^30^ observed that less than 4% of the outcome of participation was explained by the perception of barriers and that the variable explained 10% of the outcome satisfaction with life in people with spinal cord injuries. Similarly, Rochette et al.^31^ demonstrated that the perception of environmental barriers only explained a small part (6.2%) of the participation score in individuals who had suffered strokes. Dijkers et al.^24^ compared the relationship between perception of environmental barriers and social participation of people with spinal cord injury from the United States and Turkey. They found higher scores of social participation and lower scores of perception of barriers in American participants. However, such differences were attenuated when controlling for differences in age, gender, injury time, and motor repertoire. The results from that study also showed that the motor skills of participants were the main predictive factor for participation, which was minimally affected by the perception of barriers.

A recent study^3^ tested the moderating effect of environmental factors in the relationship between personal factors and the participation of children with and without disabilities in three different contexts: home, school, and community; the most pronounced effect was observed in the latter context. While the moderating effect of a variable affects the strength and/or direction of the association between two other variables, the mediating effect explains such a relationship. The results from the Anaby et al.^3^ study highlighted the mediating role of the environment, which affects the participation of children in the three contexts. Inconsistencies between the results reported by Anaby et al.^3^ and the results from the present study may be attributed to specific characteristics of the conceptual relationship tested and also to the way such concepts were operationalized. More expressive association indices might be evidenced when environmental factors and participation are anchored in the same context. The present study used the CHIEF, a general perception instrument that does not provide specific information about the perception of barriers regarding the school environment. Furthermore, the weak association detected between the perception of barriers and school participation may be explained by the fact that the relationship was examined from the perspective of negative characteristics of the environment, represented by environmental barriers. When analyzed from a positive perspective, facilitator environmental factors might show higher-magnitude relationships with the concept of participation rather than merely functioning to identify barriers. The environment in the study by Anaby et al.^3^ was examined using the questionnaire Participation and Environment Measure for Children and Youth (PEM-CY)^32^, which examined the participation in different contexts and studied the positive and negative impacts of environmental characteristics related to the participation in the context examined. The difference in results between the two studies most likely reflected the choice and specificity of the instrument used to evaluate the environmental factors.

The absence of moderation observed in the present study may also be attributed to the specificities of the sample group and the characteristics of the instrument used to evaluate the motor repertoire. The study sample exclusively consisted of individuals classified in levels I, II and III of the GMFCS so that studies that eventually also include children with higher motor impairments may show moderation results different from those reported in this study. Regarding instrumentation, the definition of the GMFCS levels included information on both motor repertoire and the walking aids used by children and youths with CP to move around their environments. Thus, the combination of information captured by the GMFCS, including the activity component and environmental factors, may have contributed to the absence of moderation because the information on environmental factors was embedded in the mobility classification of the GMFCS.

Another result observed was that the CHIEF subscale service/assistance remained significant in the regression model, even in the presence of the GMFCS. That subscale included data on transport availability, information, education and training, healthcare services and medical care, and personal and support equipment at home and in the community. Analysis of the descriptive data of the CHIEF test revealed that three of the seven items with the highest mean frequency-magnitude scores were included in that subscale (transportation, availability of education and training, and computer technology); parents identified the item transportation as the greatest barrier to participation. That result highlights the need for policies that provide access to public transportation for the disabled.

The other two environmental barriers with the highest mean scores, included in the subscale service/assistance, were availability of education and training and lack of computer technology. From the parents' standpoint, the lack of access to education or training appropriate to the needs of children was a limiting factor for the participation of their children. The lack of training geared towards the needs of children may express the teachers' lack of preparation for educating children with special needs. For teachers to be able to provide education for students with disabilities, they must know the different health conditions, capabilities, limitations, and educational needs of the students, which would then allow for the adjustment of their teaching strategies to the needs of their different students.

The subscale school/work imposed the largest barrier to participation, followed by the subscales policy and physical and structural barriers, considering the mean scores of the different CHIEF subscales observed in this study. The subscale attitude/assistance had the smallest impact on the participation of children. A comparison between those data and the data reported in the study by Law et al.^10^, who used the CHIEF to examine the perception of parents regarding the impact of environmental barriers on the social participation of their children with physical disabilities, reveals both similarities and specificities. Indeed, parents of Canadian children and youths with physical disabilities and parents of Brazilian children and youths with CP identified the subscale school/work as the greatest barrier to the participation of their children, while the subscale attitude/assistance was identified as the smallest barrier. Another interesting result when comparing both studies was that the mean values of the subscales of the present study were approximately double the values reported by Law et al.^10^; that is, the parents of Brazilian children with CP had a much higher perception of barriers to the participation of their children than the Canadian group, a result that illustrates the effect of different socioeconomic realities.

In conclusion, this study found that mobility was strongly associated with the participation of children and youths with CP. Conversely, the authors observed a small environmental impact on the school participation of those students. The hypothesis advocated by the ICF conceptual model that environmental factors significantly affect the relationship between functioning components was not supported by the present study. When moderation was examined from the negative perspective of environmental barriers, the absence of moderation suggested that general contextual factors did not change the relationship between mobility and school participation. Information on school-specific context factors may contribute to explaining the school participation of children and youths with CP.

## References

[1] Law M, Anaby D, Teplicky R, Khetani MA, Coster W, Bedell G (2013). Participation in home environment among children and young with and
without disabilities. Br J Occup Ther..

[2] Colver AF, Dickinson HO, Parkinson K, Arnaud C, Beckung E, Fauconnier J (2011). Access of children with cerebral palsy to the physical, social and
attitudinal environment they need: a cross-sectional European
study. Disabil Rehabil..

[3] Anaby D, Law M, Coster W, Bedell G, Khetani M, Avery L (2014). The mediating role of the environment in explaining participation of
children and youth with and without disabilities across home, school, and
community. Arch Phys Med Rehabil..

[4] Anaby D, Hand C, Bradley L, DiRezze B, Forhan M, DiGiacomo A (2013). The effect of the environment on participation of children and youth
with disabilities: a scoping review. Disabil Rehabil..

[5] Forsyth R, Colver A, Alvanides S, Woolley M, Lowe M (2007). Participation of young severely disabled children is influenced by
their intrinsic impairments and environment. Dev Med Child Neurol..

[6] Cury VCR, Figueiredo PRP, Mancini MC (2013). Environmental settings and families' socioeconomic status influence
mobility and the use of mobility devices by children with cerebral
palsy. Arq Neuropsiquiatr..

[7] Welsh B, Jarvis S, Hammal D, Colver A (2006). North of England Collaborative Cerebral Palsy Survey. How might
districts identify local barriers to participation for children with cerebral
palsy?. Public Health.

[8] Organização Mundial da Saúde - OMSOrganização Pan-Americana de Saúde
--OPAS (2003). Classificação internacional de funcionalidade, incapacidade e saúde.

[9] Schenker R, Coster W, Parush S (2005). Participation and activity performance of students with cerebral palsy
within the school environment. Disabil Rehabil..

[10] Law M, Petrenchik T, King G, Hurley P (2007). Perceived environmental barriers to recreational, community, and
school participation for children and youth with physical
disabilities. Arch Phys Med Rehabil.

[11] Beckung E, Hagberg G (2002). Neuroimpairments, activity limitations, and participation restrictions
in children with cerebral palsy. Dev Med Child Neurol.

[12] Kerr C, McDowell B, McDonough S (2007). The relationship between gross motor function and participation
restriction in children with cerebral palsy: an exploratory
analysis. Child Care Health Dev..

[13] Lepage C, Noreau L, Bernard PM (1998). Association between characteristics of locomotion and accomplishment
of life habits in children with cerebral palsy. Phys Ther.

[14] King G, Law M, Hanna S, King S, Hurley P, Rosenbaum P (2006). Predictors of the leisure and recreation participation of children
with physical disabilites: A structural equation modeling analysis. Child Health Care..

[15] Wiart L, Darrah J, Hollis V, Cook A, May L (2004). Mothers' perceptions of their children's use of powered
mobility. Phys Occup Ther Pediatr.

[16] Wang PP, Badley EM, Gignac M (2006). Exploring the role of contextual factors in disability
models. Disabil Rehabil.

[17] Schenker R, Coster WJ, Parush S (2005). Neuroimpairments, activity performance, and participation in children
with cerebral palsy mainstreamed in elementary schools. Dev Med Child Neurol..

[18] Palisano R, Rosenbaum P, Walter S, Russell D, Wood E, Galuppi B (1997). Development and reliability of a system to classify gross motor
function in children with cerebral palsy. Dev Med Child Neurol.

[19] Palisano RJ, Rosenbaum P, Bartlett D, Livingston MH (2008). Content validity of the expanded and revised Gross Motor Function
Classification System. Dev Med Child Neurol..

[20] Coster W, Deeney T, Haltiwanger J, Haley S (1998). School function assessment.

[21] Furtado SRC, Sampaio RF, Vaz DV, Pinho BAS, Nascimento IO, Mancini MC (2014). Brazilian version of the instrument of environmental assessment Craig
Hospital Inventory of Environmental Factors (CHIEF): translation, cross-cultural
adaptation and reliability. Braz J Phys Ther..

[22] Whiteneck GG, Harrison-Felix CL, Mellick DC, Brooks CA, Charlifue SB, Gerhart KA (2004). Quantifying environmental factors: a measure of physical, attitudinal,
service, productivity, and policy barriers. Arch Phys Med Rehabil..

[23] Han CW, Yajima Y, Lee EJ, Nakajima K, Meguro M, Kohzuki M (2005). Validity and utility of the Craig Hospital Inventory of Environmental
Factors for Korean community-dwelling elderly with or without
stroke. Tohoku J Exp Med.

[24] Dijkers MP, Yavuzer G, Ergin S, Weitzenkamp D, Whiteneck GG (2002). A tale of two countries: environmental impacts on social participation
after spinal cord injury. Spinal Cord..

[25] Associação Brasileira de Empresas de Pesquisa - ABEP (2008). Critério de Classificação Econômica Brasil.

[26] Dancy C, Reidy J (2002). Estatística sem matemática para psicologia: usando SPSS para windows.

[27] Orlin MN, Palisano RJ, Chiarello LA, Kang LJ, Polansky M, Almasri N (2010). Participation in home, extracurricular, and community activities among
children and young people with cerebral palsy. Dev Med Child Neurol..

[28] Palisano RJ, Kang LJ, Chiarello LA, Orlin M, Oeffinger D, Maggs J (2009). Social and community participation of children and youth with cerebral
palsy is associated with age and gross motor function
classification. Phys Ther.

[29] Maher CA, Williams MT, Olds T, Lane AE (2007). Physical and sedentary activity in adolescents with cerebral
palsy. Dev Med Child Neurol..

[30] Whiteneck G, Meade MA, Dijkers M, Tate DG, Bushnik T, Forchheimer MB (2004). Environmental factors and their role in participation and life
satisfaction after spinal cord injury. Arch Phys Med Rehabil..

[31] Rochette A, Desrosiers J, Noreau L (2001). Association between personal and environmental factors and the
occurrence of handicap situations following a stroke. Disabil Rehabil..

[32] Coster W, Bedell G, Law M, Khetani MA, Teplicky R, Liljenquist K (2011). Psychometric evaluation of the participation and environment measure
for children and youth. Dev Med Child Neurol..

